# The Immunology of Hepatocellular Carcinoma

**DOI:** 10.3390/vaccines9101184

**Published:** 2021-10-15

**Authors:** Gbemisola Lawal, Yao Xiao, Amir A. Rahnemai-Azar, Diamantis I. Tsilimigras, Ming Kuang, Anargyros Bakopoulos, Timothy M. Pawlik

**Affiliations:** 1 Division of Surgical Oncology, Department of Surgery, Arrowhead Regional Cancer Center, California University of Science and Medicine, Colton, CA 92324, USA; msgbemy@yahoo.com (G.L.); arahnema.azar@gmail.com (A.A.R.-A.); 2 Department of Liver Surgery, The First Affiliated Hospital, Sun Yat-sen University, Guangzhou 510080, China; xiaoyao_pumc@126.com (Y.X.); kuangminda@hotmail.com (M.K.); 3 Department of Surgery, The Ohio State Comprehensive Cancer Center, The Ohio State University College of Medicine, Columbus, OH 43210, USA; tim.pawlik@osumc.edu; 4 Department of Surgery, Attikon University Hospital, University of Athens, 12462 Athens, Greece; vincent0@otenet.gr

**Keywords:** hepatocellular carcinoma, liver cancer, vaccine, immunotherapy, immune checkpoint inhibition, PD-1/PD-L1, combined therapy

## Abstract

Liver cancer is the third leading cause of cancer death worldwide. Hepatocellular carcinoma (HCC) is the most common primary malignant tumor of the liver. Liver resection or transplantation offer the only potentially curative options for HCC; however, many patients are not candidates for surgical resection, either due to presentation at advanced stages or poor liver function and portal hypertension. Liver transplantation is also limited to patients with certain characteristics, such as those that meet the Milan criteria (one tumor ≤ 5 cm, or up to three tumors no larger than 3 cm, along with the absence of gross vascular invasion or extrahepatic spread). Locoregional therapies, such as ablation (radiofrequency, ethanol, cryoablation, microwave), trans-arterial therapies like chemoembolization (TACE) or radioembolization (TARE), and external beam radiation therapy, have been used mainly as palliative measures with poor prognosis. Therefore, emerging novel systemic treatments, such as immunotherapy, have increasingly become popular. HCC is immunogenic, containing infiltrating tumor-specific T-cell lymphocytes and other immune cells. Immunotherapy may provide a more effective and discriminatory targeting of tumor cells through induction of a tumor-specific immune response in cancer cells and can improve post-surgical recurrence-free survival in HCC. We herein review evidence supporting different immunomodulating cell-based technology relative to cancer therapy in vaccines and targeted therapies, such as immune checkpoint inhibitors, in the management of hepatocellular carcinoma among patients with advanced disease.

## 1. Introduction

Hepatocellular carcinoma (HCC) is the most common primary malignant tumor of the liver, which originates from hepatocytes [1,2]. HCC has several pathologic variants, including fibrolamellar, combined HCC-cholangiocarcinoma, clear cell, giant cell, and sarcomatoid types [1]. Chronic inflammation of the liver from cirrhosis and viral hepatitis is a well-known etiology of HCC [2,3]. However, fibrosis of the liver stemming from chronic alcohol abuse, metabolic liver disease, exposure to aspergillus fungal aflatoxin, Clonorchis sinensis infection, exposure to polyvinyl chloride, and exposure to other environmental toxins such as biphenyls, trichloroethylene, and carbon tetrachloride has also been associated with HCC development [1,2,3]. Of note, liver cancer is the third leading cause of cancer-related death, surpassed only by lung and colorectal cancers. The prognosis of HCC is most often determined based on grading and staging carried out in accordance with the American Joint Committee on Cancer’s staging system (AJCC), which takes into account the size of the tumor, local invasion of surrounding structures as well as vascular invasion and nodal status [1,2]. The Barcelona Clinic Liver Cancer (BCLC) staging system is another tool commonly used to guide the management of patients with HCC [1]. 

Surgical resection and organ transplantation remain the only potentially curative options for management of hepatocellular carcinoma [4]. Sometimes, neoadjuvant therapies are required to downstage the tumor and increase the chance of resectability [2,3]. Moreover, many patients are poor surgical candidates due to multiple co-morbidities or low functional status, requiring non-surgical management options. Hepatic artery-directed therapies, such as trans-arterial chemoembolization (TACE) and trans-arterial radioembolization (TARE), percutaneous ethanol or acetic acid injection, radiofrequency ablation, or chemotherapy using Adriamycin, carboplatin, gemcitabine are other modalities that can be considered in neoadjuvant or palliative settings when surgical intervention is not feasible [1,2]. 

Vaccination against hepatitis virus continues to be one of the most effective ways to reduce the incidence of HCC [5,6]. In view of inflammatory-based pathogenesis, there has been a growing interest in the application of immunotherapy as a systemic therapy in management of patients with HCC. In 2008, administration of sorafenib, a multi-kinase inhibitor that targets vascular endothelial growth factor (VEGF), platelet-derived growth factor (PDGF), and rapidly accelerated fibrosarcoma (RAF) in advanced HCC was demonstrated to improve overall survival (OS) and time to progression (TTP) compared with placebo [7,8,9]. However, even with application of sorafenib as a first-line treatment and lenvatinib, regorafenib, cabozantinib, ramucirumab as second-line treatment options, the disease control rates remain less than 60%, with an objective response rate less than 10% [7,10]. 

Despite recent advances in the management of hepatobiliary malignancies, the incidence of HCC continues to rise globally with increased mortality in advanced stages [5,6]. We herein review the role of immunomodulating cell-based technology in the development of vaccines, as well as immune checkpoint inhibitors administered in the management of patients with hepatocellular carcinoma. 

## 2. Landscape of the Immune Microenvironment in HCC

**Figure 1:**
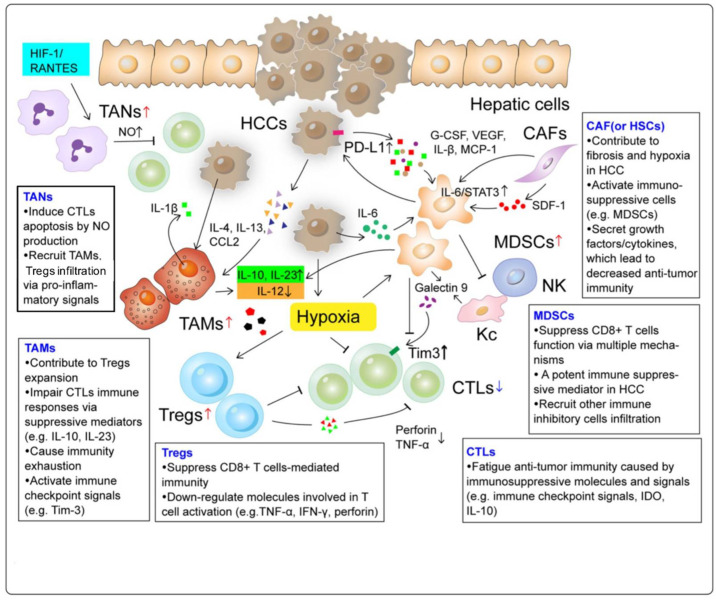
The landscape of an immunosuppressive tumor microenvironment.

**Figure 2:**
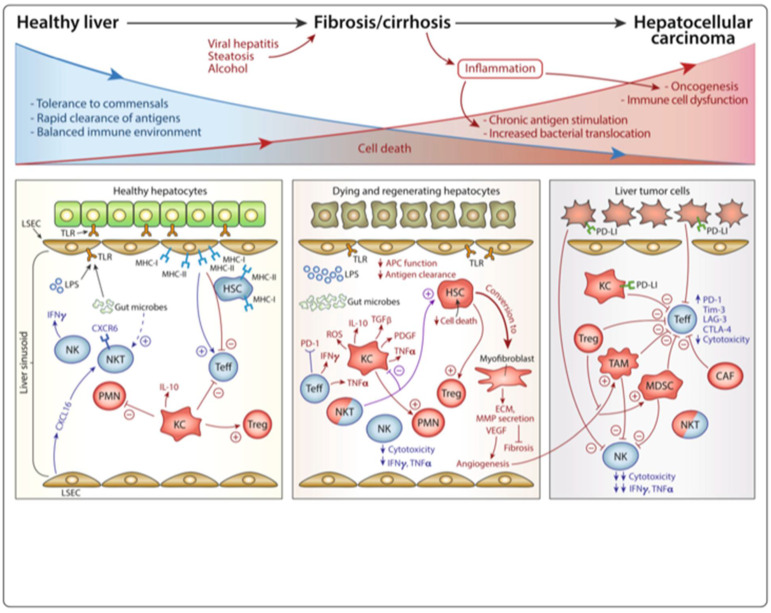
Liver immunobiology across a spectrum from healthy liver to inflammation and oncogenesis.

The immune microenvironment of the liver is a multicomponent system consisting of hepatocytes, immune cell subsets, immune receptors and ligands, cytokines, and chemokines, extracellular matrix, and other elements (Figure 1) [11,12,13]. In a healthy liver, this complex network is delicately regulated to maintain a dynamic balance between tolerance and immunity. When chronic inflammation is induced by some infectious or pathogenic agents, both the innate and adaptive immune systems can be altered, leading to tumorigenesis (Figure 2) [14,15]. 

### 2.1. Tumor-Associated Macrophages (TAMs) 

TAMs are one of the most abundant tumor-infiltrating immune cell types containing two polarizing phenotypes of tumor suppressive M1 and oncogenic M2. After blocking the IL-6/STAT3 pathway, HCC cells co-cultured with macrophages exhibit cell apoptosis, reduced drug resistance, suppressed cell invasion, migration, and tumor formation; all are indicators of the enhanced tumor suppressive function of M1 [16,17]. The M2 phenotype stimulates tumor initiation, progression, and metastasis by various mechanisms involving TLR4/STAT3, TLR4/TRIF/NF-κB, Wnt/β-catenin, and many other pathways [18]. The crosstalk between TAMs and other cell types such as MDSCs and Tregs generates a series of changes in chemokine production, MHCI/II expression, and downstream T cell activation, which are correlated with immunosuppression and HCC development [13]. 

### 2.2. Tumor-Associated Neutrophils (TANs)

TANs are neutrophils that infiltrate into the tumor microenvironment. TANs are activated and modulated by various molecules, such as IL-17, IFN-β, CXCL1, CXCL2, and CXCL5 [19]. Similar to TAMs, TANs can be classified as antitumor N1 or protumor N2 phenotypes. The tumorigenic role of neutrophils recruited to the inflammatory sites may be attributed to multiple mechanisms. The loss of hypoxia-associated factor (HAF) results in upregulation of the HIF-1/RANTES pathway and accumulation of infiltrating TANs, which is associated with HCC initiation and progression from non-alcoholic steatohepatitis [20]. Examination of HCC clinical samples have revealed that the accumulation of infiltrating TANs in the peritumoral region mediates the overexpression of PD-L1 and is negatively correlated with T cell abundance. In addition, a decreased neutrophil-to-lymphocyte T cell ratio in peritumoral tissue (pNLR) has been correlated with prolonged patient survival after surgical treatment, suggesting the potential prognostic values of TANs [21]. 

### 2.3. Myeloid-Derived Suppressor Cells (MDSCs)

MDSCs are heterogeneous populations of early myeloid progenitors and immature granulocytes at various stages of differentiation. MDSCs have a suppressive capacity both on innate immune activities of NK cells and adaptive immune responses by CD4+ and CD8+ T cells [13]. Multiple pathways are responsible for MDSC-mediated T cell suppression in the HCC microenvironment. Arginase 1 (ARG1), inducible nitric oxide synthase (iNOS), and reactive oxygen species (ROS) produced by MDSCs can inhibit CIK cell cytotoxicity against HCC [19]. Together with other secreted immunosuppressive cytokines, such as IL-10 and TGF-β, these molecules allow MDSCs to inhibit T cell proliferation, dendritic cell development, cytotoxic T cells and NK cell functioning, as well as stimulate Treg expansion. The Tim-3/Gal-9 pathway regulates Th1 immune responses directly by triggering cell death in Th1 cells and indirectly by expanding a granulocytic MDSC subtype, leading to a downregulation of IFN-γ production and T cell apoptosis [22]. 

### 2.4. Regulatory T Cells (Tregs)

Tregs are a subpopulation of T cells with a suppressive regulatory influence on the immune system. Tregs are activated by the interaction between T cell receptors (TCR) and its ligands, as well as the IL-10 and TGF-β signaling pathways. Tregs can inhibit T cell proliferation and cytokine production and play an important role in preventing an autoimmune response. A CD4^+^CD25^+^Foxp3^+^ Treg subset inhibits the killing ability of CD8^+^ T cells by blocking the production and release of perforin and granzymes, as well as suppressing certain cytokines that are necessary for CD8^+^ T cell activation, such as TNF-α and IFN-γ [23]. Apart from functioning on cytokine release, long noncoding RNAs (lncRNAs) also play a role in driving Treg differentiation during HCC progression [24]. 

## 3. Immunotherapy

### 3.1. Indirect Therapy

Understanding the mechanism of adaptive immunity was an important step in establishing cancer immunotherapy. The adaptive immune response is one of the main protective mechanisms of the human body against foreign antigens, including cancer cells. After identification of cancer cells, immune cells such as dendritic cells (DCs), known as professional antigen presenting cells (APCs), capture and process these tumor-associated antigens (TAAs). DCs, which are present in the blood, peripheral and lymphoid tissues, activate a robust immune response including chemokines and mediators of inflammation in order to curtail the extent of an infection in the body. DCs achieve this goal by activating lymphocytes, including T cells, B cells, natural killer (NK) cells and NK T cells. DCs also migrate to regional lymph nodes, where they generate antigen-specific immune responses. The level of specificity seen with DCs is mainly attained by the presence of different DC subtypes in different anatomical locations of the body. For example, there are at least two subsets of DCs: myeloid DCs and plasmacytoid DCs in the blood stream. In the skin, myeloid DC subsets are further categorized into epidermal Langerhans cells (LCs) and dermal (interstitial) DCs. Each subtype has its own distinct specific role [7,8]. Due to the high specificity involved in this process, vaccine therapies have proven efficiency while maintaining a low toxicity profile, making them potentially favorable in the prevention and management of HCC [25]. 

#### 3.1.1. Vaccines

##### Oncolytic Virus Vaccines

Vaccination in cancer therapy happens through in vivo delivery of specific TAAs, tumor lysates, and nontumor-associated individual antigens. These vaccine antigens are then processed by APCs such as DCs, and then coupled to MHC class I and II molecules to activate CD8 cytotoxic T lymphocytes and CD4 helper lymphocytes [10]. 

An example of a vaccine-driven cancer therapy is the use of a recombinant tumor lysate, Hep G2 cell line lysate, to target alpha-fetoprotein (αFP). Human αFP is present in up to 80% of HCCs and is commonly used as a biomarker for diagnosis and surveillance of HCC. A clinical trial conducted by Zhu et al. noted how αFP can also be used as a prospective target for immunotherapy in HCC. Zhu et al. used lentivector and peptide immunization to develop genetically HLA-A2 modified CD8 T cells in transgenic mice capable of recognizing a specific epitope of αFP (αFP 158). Five million HepG2 cells were inoculated into immunocompromised non-obese diabetic severe combined immunodeficient gamma knockout (NSG) mice. The transfer of αFP 158 epitope-specific CD8 T-cell cells was demonstrated to eradicate HepG2 tumor xenografts of approximately 2 centimeters and above in the NSG mice. One weakness of oncolytic virus vaccines highlighted in the study was that murine TCRs had a high affinity for human antigenic epitopes, thereby increasing the risk of cross-recognizing off-target antigens, resulting in toxicity. The authors suggested that a more diverse TCR repertoire is needed to select the optimal TCRs that can provide tumor-killing activity without toxicity to normal human T cells [26].

##### Dendritic Cells Vaccines

Dendritic cells are the most important APCs in the body, with a vital role in the activation of the immune system, leading to T-cell differentiation. In a phase I/II clinical trial, mature DCs charged with HCC antigens such as αFP, glypican-3 (GPC-3) and melanoma antigen gene-1 (MAGE-1) were administered as a vaccine to patients with advanced HCC. Vaccines were well tolerated by all patients with no grade III/IV adverse events. All patients demonstrated a strong T cell response against HCC, with a high reactivity against αFP and moderate reactivity against GPC-3 and MAGE-1. Compared with the control group, 13.3% and 60% of patients treated with dendritic cells pulsed with HCC antigens had a radiologic regression and disease stabilization, respectively. According to this study, the DC vaccine was well tolerated. Only mild toxicity was experienced by participants in the form of an injection site reaction and fever; otherwise, there were no hematological, hepatic, or renal toxicities [27].

##### Antigen Peptide Vaccines

Protein antigens have also been recognized as targets in vaccine immunotherapy. GPC3, αFP, NY-ESO-1, SSX-2, human telomerase reverse transcriptase (hTERT), hepatocellular carcinoma-associated antigen-587 (HCA587), and melanoma antigen gene-A (MAGE-A) are some examples of such protein antigens [25]. 

In one study, injection of a dendritic cell line of tumor-bearing mice with a lentivirus expressing the murine DEX-αFP gene weekly for three weeks was associated with significantly slower tumor growth [28]. Additionally, a longer survival rate was noted in 100% of DEX-αFP mice [28]. A phase I study was conducted using GPC3 in patients with advanced HCC who were treated with conventional therapies such as surgery, radiofrequency ablation, TACE, and radiation therapy. Five patients received a HLA-A2-restricted GPC3 peptide, and 6 patients received a HLA-A24-restricted GPC3 peptide. Over an average follow-up of five months, the vaccine therapy was well tolerated, with a resultant proliferation of TAA-specific cytotoxic T lymphocytes targeting cancer cells. At one year, patients who received a combination therapy of surgery and vaccination had a two times lower recurrence rate (RR) compared with patients who only underwent surgery (RR of 24% in combination group; *p* = 0.047). At two-year follow-up, the recurrence rates in the combination therapy group and the surgery-only counterparts were 52.4% versus 61.9%, respectively (*p* = 0.387) [29]. Similar to the adverse reactions described above with DC vaccines, study participants also experienced a skin reaction with the GPC3-peptide vaccine therapy, yet this therapy was otherwise very well tolerated.

#### 3.1.2. Immune Checkpoint Inhibitors 

There are many processes in place to curtail and prevent an overactivation of T cells. Examples of regulatory molecules involved in such inhibitory mechanisms include programmed cell death 1 (PD-1), programmed cell death ligand 1 (PD-L1), and cytotoxic T lymphocyte–associated antigen 4 (CTLA-4) [30]. 

##### Programmed Cell Death-1 (PD-1) Inhibition 

PD-1 is expressed in a wide range of immune system cells, including activated CD8 and CD4 T-lymphocytes, as well as B cells, natural killer cells, T-regulatory cells, monocytes, and dendritic cells. The PD-1 ligands are PD-L1 found in hematopoietic and parenchymal cells, while PD-L2 are found only in hematopoietic cells. Upregulation of PD-1 ligands by cytokines inhibits immune cell activation and leads to T cell exhaustion. Cancer cells use these ligands to suppress immunosurveillance [31]. Atezolizumab, pembrolizumab and nivolumab are some examples of PD-1 and PD-1 ligand inhibitors. 

In another study, T cells were sorted by flow cytometry and the presence of a specific transcriptional factor, thymocyte selection-associated high mobility group box (TOX) protein, was measured in T cells. The role of TOX is not clearly defined; however, it was noted that an abundance of TOX protein expression in CD8 T cells was associated with a high level of PD-1 ligands in HCC, leading to the suppression of T cell tumor fighting abilities by means of a reduction in PD1 degradation and promotion of PD1 translocation to the CD8 T cell surface. As a result, downregulation of TOX proteins can lead to an improvement in the anti-tumor function of CD8 T cells. In such cases, antibodies against immune checkpoints such as PD1 can be used to prevent the exhaustion of CD8 T cells expressing high levels of PD1 markers. A limitation of this therapy is that PD1 expression in cancer cells does not correlate with PD1 blocker response in patients with HCC (i.e., PD1 expression was not an accurate biomarker of response to anti-PD1 therapy by itself) [32]. 

##### Cytotoxic T Lymphocyte–Associated Antigen 4 (CTLA-4) Inhibition

CTLA-4 is an immune checkpoint molecule expressed on T cells with an inhibitory effect on T cell activation. CD80 and CD86 are B7-costimulatory ligand molecules that activate T cells when they interact with CD28. CTLA-4 blocks the interaction of CD28 with these co-stimulators of T cells to suppress T cell upregulation [33]. The efficacy of CTLA-4 inhibition against melanoma has been well studied. The effect of CTLA-4 inhibition against HCC was studied in a phase 2 study using anti-CTLA4 (tremelimumab) plus tumor ablation therapy [34,35]. In this study, 39 patients with HCC were given tremelimumab by IV every 4 weeks for a total 6 doses, followed by 3-monthly administrations until off-treatment criteria were met. On day 35, patients received subtotal radiofrequency ablation (RFA) or chemoablation. Tremelimumab therapy caused an increase in T cell responses and led to identification of biomarkers in the peripheral blood of the treated patients, which may serve to track regression of disease, as well as progression in patients responding to anti-CTLA4 immunotherapy. A limitation of this therapy was that there is currently no biomarker expression measurement that can be used as a reliable predictor of responsiveness to immune checkpoint therapy across multiple cancers [34,35].

### 3.2. Direct Therapy

Adoptive cell therapy (ACT) is a type of immunotherapy in which the patient’s own immune cells (usually T cells) are modified in vitro and reinfused into the patient body to exert antitumor, antiviral, or anti-inflammatory effects. One potential mechanism of immune escape and tumor progression in HCC is the pro-immunosuppressive modulation of MHC-I and MHC-II expression on the cell surface, as well as the lack of HCC-specific antigens to allow T cells to recognize and distinguish tumor cells from normal hepatocytes. ACTs, however, aim at enhancing the ability of T cells to recognize and present the tumor-specific antigens and activate immune elimination, either dependent or independent of MHC expression [36]. Currently, there are several forms of ACT developed for cancer treatment: chimeric antigen receptor (CAR) T cells, cytokine-induced killer (CIK) cells, T cell receptor (TCR) T cells, tumor-infiltrating lymphocytes (TILs). 

#### 3.2.1. CAR-T Cells

The antigen-binding domain in a CAR is a single-chain variable fragment (scFv) derived from the variable regions of a tumor-specific monoclonal antibody and is made up of a heavy and a light chain connected with a short linker. When bound to the external antigen, an activation signal is transmitted to the intracellular signaling domain and induces perforin- and granzyme-mediated apoptosis [37]. 

GPC-3 is highly expressed in HCC and has been shown to correlate with a poor prognosis. GPC-3 is the most commonly used HCC-associated antigen for CAR-T. Besides the efficacy of GPC3-targeted CAR-T cells to kill GPC-3-positive HCC cells in vitro, they can eradicate HCC xenografts that highly express GPC-3 and suppress the growth of tumors in vivo [38,39]. Another TAA under investigation is NKG2D, which efficiently eliminates HCC cell lines SMMC-7721 and MHCC97H [40]. Many strategies have been designed to deal with the unpleasant on-target/off-tumor toxicities. One way to reduce toxicity is to add a second recognition site to construct a dual-targeting CAR, consequently requiring double-positive antigen expression on the target cells to increase the specificity. Development of GPC-3/EGFR, GPC-3/ASGR1, and αFP /MHC complex CAR-T cells has been associated with improved activation, expansion, and persistence of the T cells, as well as stronger cytotoxicity in double-positive tumor cell groups [41,42,43]. An attempt to design CD39^+^ CAR-T cells targeting the HBV surface protein demonstrated increased secretion of IFN-γ and promising anti-tumor activity, especially with PD-1 knockdown [44]. Zhang et al. designed a novel inducible CD147-targeted CAR-T system in which the on-off expression of CD147 can be controlled by the addition of doxycycline under certain desired situations, facilitating decreased toxicity and adverse effects [45]. Tseng et al. devised a logic-gated GPC3-synNotch-inducible CD147-CAR-T system capable of selectively attacking GPC3^+^CD147^+^ HCC cells in transgenic mouse models with minimized on-target/off-tumor toxicity [46]. 

The data of two sequential first-in-human phase I clinical trials (NCT02395250, NCT03146234) of GPC3-CAR-T cell therapy for patients with refractory or relapsed HCC has been published. In a total of 13 patients receiving autologous GPC3-CAR-T cells following fludarabine and cyclophosphamide, there were 2 objective responses and 1 patient with stable disease who also achieved long-term survival of 44.2 months. These patients all had high-percentage reductions in serum αFP level. The OS at 6 months, 1 year and 3 years were 50.3%, 42.0% and 10.5%, respectively, with a median OS duration of 39.7 weeks [47]. Currently, there are eight trials of CAR-T therapy for HCC recruiting patients, among which five are targeting GPC-3, one targeting CD147, and the other two are multi-targeted. 

#### 3.2.2. CIK Cells

CIK cells are a group of heterogeneous CD3^+^ effector T cells mainly populated with two predominant subsets, NKT-like (CD3^+^CD56^+^) and cytotoxic T-like (CD3^+^D56^-^) cells. CIK cells are generated from extracted human PBMCs or cord blood mononuclear cells. After ex vivo incubation and stimulation with cytokines such as IFN-γ, anti-CD3 antibody, IL-1 and IL-2, they complete maturation and are transfused to the recipients. CIK cells can recognize tumor cells in an MHC-unrestricted manner; thus, they are particularly effective in tracking and attacking tumor cells missing MHC markers [48]. 

In 2002, Wang et al. generated CIK cells from healthy donors and patients with primary HCC and described the proliferating curve and antitumor characteristics in vitro and in vivo [49]. The cytotoxicity of CIKs against a selected multi-drug resistant HCC cell line Bel-7402 was above 50% [50]. By using in vivo bioluminescence imaging in tumor xenograft-bearing mice, it was verified that CIK cells kill stem-like HCC cells via NKG2D-ligand recognition [51]. DC-CIK cell co-cultivation also possesses high antitumor ability against HCC, especially αFP -expressing HCC cells [52,53]. 

According to recently reported data on DC/CIK-CD24, the four-year survival of patients receiving two or four doses of DC/CIK-CD24 after radical resection were 47.1% and 52.6%, respectively [54]. A meta-analysis involving 22 studies of 3756 patients demonstrated that DCs and/or CIKs combined with conventional therapy significantly improved OS at 6 months (RR = 1.07; 95% CI: 1.01–1.13, *p* = 0.02), 1 year (RR = 1.12; 95% CI: 1.07–1.17, *p* < 0.00001), 3-years (RR = 1.23; 95% CI: 1.15–1.31, *p* < 0.00001) and 5 years (RR = 1.26; 95% CI: 1.15–1.37, *p* < 0.00001) [55]. High numbers of PD-1 positive intra-tumor lymphocytes, CIK cell cytotoxicity and serum AFP were suggested to serve as prognostic factors to predict clinical outcomes related to CIK as an adjuvant therapy [56,57,58]. 

#### 3.2.3. TCR Engineered T Cell Therapy

The recognition of antigens presented by MHC is mainly achieved by the α and β chains binding with a CD3 complex, which together constitute the TCR. By screening and isolating T cells with high affinity to tumor-specific antigens from PBMCs, the sequences of α and β chains of these TCRs are obtained. After inserting the desired TCR gene sequences into human T cells and expanding them ex vivo, these stable genetically engineered T cells can be reinfused to exert an antitumor function. 

The HLA-A∗02:01-restricted nonapeptide HBs_371-379_-ILSPFLPLL was targeted by the Ai-TCR-T system, whose potency to recognize a range of HBV variants and eliminate HCC cells has been verified [59]. Docta et al. used a combination of physiochemical and cell biological methods to construct an optimized TCR with improved affinity to αFP /HLA-A∗02^+^ tumors, laying the foundation of the later first-in-human trial of HCC adoptive T cell immunotherapy (NCT03132792). In this trial, in four patients with HLA-A∗02:01^+^ or 02:642^+^ and certain αFP expression levels who received the treatment, one patient had a complete response, one had stable disease, and two had progressive disease [60,61]. Luo et al. identified a TCR recognizing the HLA-A∗02:01-restricted αFP _158−166_ peptide FMNKFIYEI and have initiated a phase I clinical trial among patients with an HLA-A∗02:01 positive allele [62]. 

#### 3.2.4. TILs

TILs are a mixed group of immune cells isolated from tumor samples or peritumor stroma. Due to strong exposure to tumor cells, TCRs in TILs presumably gain the ability to recognize a broad range of antigens and achieve high antitumor efficiency. According to recent findings, different cell components of TILs may have different roles. For instance, NK and CD8^+^ T cell densities are positively correlated with tumor apoptosis and predict longer survival, while high levels of FoxP3^+^ TILs indicate worse OS and RFS in HCC patients [63,64]. The overall efficiency of TILs in HCC tumor progression control depends on the expression level of immune inhibitory receptors. An attempt to incubate patient TILs with antibodies against PD-1, TIM3 or LAG3 can restore TIL proliferation and cytokine production [64]. In a phase I clinical trial, 15 out of 17 recruited HCC patients after surgery had their TILs expanded and activated for refusion. With a median follow-up of 14 months, all patients were alive, 12 patients had no evidence of disease, and the other 3 had tumor recurrence [65]. The main limitation of ACT studies is that the clinical trials are restricted to Asia and fail to involve data from other regions. In addition, the lack of appropriate criterion and standard informed consent have raised concerns of over-commercialization. 

A major risk and direct cause of adverse effects of ACT such as CAR-T or TCR engineered T-cell therapy in preclinical and clinical trials is the on-target, off-tumor toxicity. Many tumor antigens are also expressed on normal cells in essential tissues and require specific measures to counteract potential toxicities resulting from their off-tumor recognition [66]. Both for CAR-T cells and other ACTs, the choice and validation of the target antigen is of great importance for optimal clinical efficacy and minimization of on-target/off-tumor side-effects. Another common complication is tumor lysis syndrome (TLS), which is a result of the release of the cellular contents of killed tumor cells into the bloodstream. The abrupt changes in blood electrolytes and metabolites can ultimately result in acute uric acid nephropathy, acute kidney failure, seizures, cardiac arrhythmias, and death [67]. Some clinical trials have also reported cytokine release syndrome (CRS) after CAR-T therapy. Following the activation of the CAR-T cells, a variety of inflammatory cytokines are released, including IFN-c, TNF-a, IL-1b, IL-2, IL-6. The released cytokines can trigger an acute inflammatory response and induce endothelial and organ damage, leading to microvascular leakage, heart failure and even death [68]. A special concern of microbiological contamination was suggested. Microbiological contamination, such as bacteria, viruses, or endotoxins, may enter during blood collection from a patient or during CIK cell cultivation. Therefore, a series of blood tests for various pathogens before blood collection is necessary, and patients with positive results will require an isolated culture environment [48].

## 4. Combination Immunotherapy

Despite initial encouraging results, studies investigating the efficacy of monotherapy regimens using TKI such as sorafenib have not been promising. Many patients with advanced HCC continue to experience disease progression with a relatively short progression-free survival leading to a high rate of resistance [69]. Therefore, combination therapy has gained increasing interest due to its efficiency in overcoming primary resistance (Table 1). 

### 4.1. Dual Immune Checkpoint Inhibitors—Combined PD-1 and CTLA-4 Inhibition

In CheckMate-040, a multicenter and multicohort phase Ib/II trial, the efficacy of combination therapy with nivolumab, a PD1-inhibitor, and ipilimumab, a CTLA-4 inhibitor, was studied. A total of 148 patients were randomized to Arm A (nivolumab 1 mg/kg plus ipilimumab 3 mg/kg, administered every 3 weeks (4 doses), followed by nivolumab 240 mg every 2 weeks), Arm B (nivolumab 3 mg/kg plus ipilimumab 1 mg/kg, administered every 3 weeks (4 doses), followed by nivolumab 240 mg every 2 weeks), and Arm C (nivolumab 3 mg/kg every 2 weeks plus ipilimumab 1 mg/kg every 6 weeks). Patients in Arm A were reported to have higher rates of adverse events, yet also had the most promising survival (median OS = 23-month, 95% CI, 9-NR, favorable ORR (32%)). Grade 3–4 toxicity was observed in 37% of patients. The authors concluded that combination regimen was safe with manageable adverse effects. As a result, combination therapy with nivolumab and ipilimumab received accelerated approval in the US as a second-line therapy for HCC. Consideration of the therapy as first-line is currently being investigated [71,72]. In a phase I/II open-label randomized study of patients with advanced HCC, Kelley et al demonstrated that combination therapy with durvalumab, a PD-L1 inhibitor, and tremelimumab, a CTLA-4 inhibitor, was associated with ORR of 17.5%; 7 out of 40 evaluable patients had a partial response [39]. No unexpected safety concerns were observed, while the most common grade ≥3 related adverse effect was an asymptomatic increased AST. As a result, the study has progressed to a randomized phase III study (HIMALAYA trial) in order to assess the efficacy of durvalumab and tremelimumab combination therapy versus sorafenib monotherapy as a first-line treatment for patients with treatment-naïve advanced HCC [73]. 

### 4.2. NK Cells and Immune Checkpoint Inhibitors

Natural killer (NK) cells are innate immune cells that use the specific cell receptors CD56 and CD16. A large amount of NK cells can be found in the liver sinusoids; additionally, NK cells constitute approximately 30% of liver lymphocytes, which makes them a good target for HCC immunotherapy [74]. NK cells in the liver are maintained by MHC-1 inhibitory receptor molecules such as killer cell immunoglobulin-like receptors (KIR) and CD94/NKG2A [74]. NCT01714739 is the subject of an ongoing phase I/II clinical trial studying the efficacy of the anti-KIR antibody lirilumab combined with an anti-PD1 antibody nivolumab, and nivolumab plus anti-VTLA4 antibody ipilimumab in patients with advanced refractory solid tumors [75]. In another ongoing phase II trial (NCT02643550), combination therapy of monalizumab, an anti-NKG2A antibody, with cetuximab, an anti- EGFR, in patients with head and neck squamous cell carcinoma was associated with an objective response rate of 31%. Such promising results may inspire clinical trials specifically studying the use of NK cell inhibition in dual immunotherapy against HCC [76]. 

### 4.3. Targeted Therapies and Immune Checkpoint Inhibitors—Combined VEGF and PD-1 Inhibition

HCC relies on a supply of nutrient and oxygen by means of angiogenesis and vascular permeability through VEGF. The relationship between VEGF expression and HCC progression is directly proportional [77]. Sorafenib is a multi-kinase VEGF inhibitor that acts as multi-targeted immunomodulator, inhibiting cell surface tyrosine kinase, as well as intracellular serine-threonine kinase. Sorafenib suppresses angiogenesis and tumor cell proliferation by inhibiting the Ras/Raf/MEK/ERK signaling pathways, as well as the platelet-derived growth factor receptor (PDGFR), vascular endothelial growth factor receptor (VEGFR), and hepatocyte factor receptor (c-KIT) [78]. The efficacy of sorafenib has been recorded over the past years; however, usage has been limited due to its cytotoxic effects leading to severe adverse effects, including worsening hepatic function [77,78]. In CheckMate-459, a multicenter phase 3 trial, 743 patients with advanced HCC were randomized to receive nivolumab, a PD1-inhibitor or sorafenib, a tyrosine kinase VEGF inhibitor, as a first-line treatment. While OS did not meet the predefined threshold of statistical significance, the nivolumab group did record an improvement in OS, objective response and complete response rates with a promising safety profile [79]. 

IMbrave150 is a multicenter phase 3 randomized trial designed to study the efficacy and safety of atezolizumab, an anti–PD-L1, in combination with bevacizumab, an anti-VEGF, compared with sorafenib in patients with advanced or unresectable disease who have received no prior systemic therapy. Atezolizumab and bevacizumab combination therapy delayed deterioration of quality of life compared with sorafenib. There was also a statistically and clinically significant improvement of OS (HR, 0.58, 95% CI, 0.42–0.79), progression-free survival (PFS) (HR, 0.59, 95% CI, 0.47–0.76), and higher ORR (odds ratio, 2.77, 95% CI, 1.62–4.74). Treatment toxicity was comparable between two groups with grade 3–4 TRAEs of 56.5% versus 55.1% in the combination therapy group compared with the sorafenib arm [80]. In the aftermath of this trial, combination immunotherapy with atezolizumab and bevacizumab received FDA approval as a first-line therapy for HCC [80]. KEYNOTE 524 is a phase I clinical trial evaluating the efficacy of a combination therapy using lenvatinib, a multiple receptor tyrosine kinase inhibitor, plus pembrolizumab, an anti-PD-1 therapy, in patients with advanced unresectable HCC with no prior systemic therapy. The primary endpoints were objective response rates (ORR) and duration of response (DOR) by modified Response Evaluation Criteria in Solid Tumors (mRECIST) and RECIST. In patients treated with the combination therapy, the ORR was 36% (95% CI: 26.6–46.2), with a complete response rate of 1% and a partial response rate of 35%. The median DOR was 12.6 months (95% CI: 6.9-not estimable) [81]. A phase 1b LIVER 100 trial has examined the use of a combination therapy including avelumab, an anti–PD-L1 IgG1 antibody, and axitinib, a tyrosine kinase inhibitor selective for VEGF receptors 1, 2 and 3. The ORR was 13.6% and median PFS was 5.5 months when evaluated by RECIST; ORR was 31.8% and median PFS was 3.8 months based on mRECIST. The most common grade 3 TRAEs were hypertension (50.0%) and hand-foot syndrome (22.7%), and there was no evidence of any grade 4/5 TRAEs [82]. 

### 4.4. Locoregional Therapies and Immune Checkpoint Inhibitors

Locoregional therapies, including hepatic artery-directed therapies, percutaneous injection, and ablation, may boost the immune system response to cell death caused by local ischemia and cytotoxicity generated by locoregional therapy [74,83]. In a single arm phase I/II trial study evaluating the effects of tremelimumab, a CTLA-4 inhibitor combined with ablative therapies, thirty-two patients with advance HCC received 3.5 and 10 mg/kg tremelimumab IV every 4 weeks for a total of 6 doses followed by 3-monthly maintenance doses. During induction, at day 36, patients underwent subtotal radiofrequency or chemo-ablation in order to activate a collaborative immunogenic cell death. Out of 19 evaluable patients, five patients (26%) achieved a partial response. Median time to tumor progression was 7.4 months (95% CI 4.7 to 19.4 months) and median OS was 12.3 months (95% CI 9.3 to 15.4 months). Tissue biopsies obtained at six weeks revealed an increase in CD8^+^ T cells intratumorally, with patients having a clinical benefit. This combination therapy was generally safe; the most common adverse reaction was grade 1 pruritus and rash consistent with immune-related dermatitis. One patient was noted to have grade 2 persistent diarrhea secondary to colitis. Another developed grade 2 autoimmune pneumonitis, which resolved after the patient was taken out of the study [84]. A few ongoing phase II studies are investigating the efficacy of nivolumab plus pembrolizumab in combination with conventional radioembolization (NCT03397654 and NCT0314370), stereotactic body radiation therapy (SBRT) (NCT03316872), and Y90 radioembolization (NCT03099564), as well as nivolumab with Y90 (NCT03033446) in the management of patients with HCC [85]. 

### 4.5. Bispecific Antibodies

Bispecific antibody (BsAb) therapy is the combination of two or more antibodies into one immunotherapy agent. In BsAb therapy, while one antibody activates the effector T cell receptors such as CD3 and CD28, the other antibody causes a tumor-associated antigen, such as AFP, GPC3, and EpCAM, to initiate tumor cytotoxicity [86]. 

NCT02748837 is a multi-center phase I study conducted to assess the efficacy of ERY974 in glypican3 (GPC 3)-positive HCC. A combination of anti-GPC 3 and anti-CD3 antibodies was used to form an IgG4 bispecific antibody called ERY974 for patients with advanced solid tumors. The bispecific therapy, ERY974, proved to be effective in a variety of mouse cancer models, even in large, advanced stage tumors and tumors with nonimmunogenic features. Toxicity experienced with this therapy was an asymptomatic transient elevation in cytokines; no organ toxicity was evident [87]. NCT03517488 studies the use of anti-PD-1 and anti-CTLA-4 to reactivate tumor lymphocytes in advanced HCC. NCT03752398 is another trial which evaluates the efficacy of bispecific antibody therapy by targeting PD-1/ICOS in subjects with advanced HCC. NCT03517488 and NCT03752398 are still ongoing; however, bispecific antibody therapy proves promising, as it has shown substantial antitumor activity in mouse HCC.

## 5. Potential Prognostic Biomarkers for Immunotherapy

Despite a growing number of studies on HCC immunotherapeutic strategies, the treatment effect and patient outcomes appear to vary among different subgroups due to significant etiologic and molecular heterogeneity. Therefore, development and validation of prognostic biomarkers have become increasingly crucial to guide immunotherapy. Application of tumor PD-L1 expression as an indicator for anti-PD1 therapy has been approved in some cancer types; however, results of clinical trials for its predictive value in HCC have been contradictory. Lack of a standard cutoff, using a single evaluation for a dynamic process, incompatibility among different types of assays, and induced PD-L1 expression by previous treatment are some of the reasons for such inconsistency. Furthermore, a combined positive score of PD-L1 expression in immune and tumor cells has been associated with response to pembrolizumab [88,89]. Serum PD-1 (sPD-1) or PD-L1 (sPD-L1) were discovered to be favorable and negative independent prognostic factors of DFS and OS in HCC patients, respectively. However, their association with patient response to anti-PD-1 therapy warrants further investigation [90]. In the tumor microenvironment, an increase in CD8^+^ cell infiltration and two other clusters recognized by CyTOF analysis were shown to correlate with response to immune checkpoint inhibitory treatment. The CD8^+^ T cell/Treg ratio calculated among these clusters also increased after treatment, while the ratio in nontumor tissue demonstrated no significant change [91]. 

Tumor mutational burden (TMB), defined as the number of somatic nonsynonymous mutations per mega-base in tumor cells, correlates with favorable outcomes with anti-PD-1 treatment among various types of tumors, such as melanoma and NSCLC [92]. An extensive literature research plotted the objective response rates against TMB in various tumor types and demonstrated a correlation of the median TMB level (5 mutations/Mb) in HCC and the objective response rate (17.5%) [93]. A case series study investigated the TMB level in 17 patients with advanced HCC who received PD-1 inhibitors and revealed that TMB levels showed no significant variation between patients with stable or responsive disease (ranging from 3–15 mutations/Mb) versus patients with progressive disease (ranging from 4–9 mutations/Mb) [94]. Tumors with deficient mismatch repair (dMMR) or high-microsatellite instability (MSI-H) contain high levels of lymphocyte infiltrates and exhibit strong expression of immune checkpoints, including PD-1 and PD-L1. It has been recognized that a large group of MMR-deficient cancers are sensitive to immune checkpoint blockades. dMMR is found, however, in only less than 4% of HCC patients, while MSI-H is observed in less than 3% of cases [95,96,97]. Overall, TMB and dMMR/MSI-H are promising prognostic indicators for immunotherapy in certain types of solid tumors but are considered either not significant between subgroups or infrequent in HCC and thus require more evidence to serve as predictive factors for HCC. 

The increasing utility of next-generation sequencing in patients with various tumors may help identify potential predictive biomarkers. The Wnt pathway activation is associated with T-cell exclusion and innate resistance to immune checkpoint blockade in vivo. In a cohort of 31 patients receiving immune checkpoint inhibitors, compared with the non-Wnt pathway altered group, patients with Wnt-activated HCC had shorter median PFS and median OS [98]. Studies exploring alterations in epigenetic factors have revealed that circRNAs play an important role in the regulation of tumor proliferation, migration, metastasis, immunosuppression, and treatment resistance. Retrospective data from 30 HCC patients who received anti-PD-1 therapy demonstrated that circUHRF1 expression level is much higher in the patients with progressive disease versus individuals with partial response or stable disease. The number of NKG2D-positive cells in this group was also significantly reduced, indicating that NK cell activation and functions are attenuated. In this study, the implantation of circUHRF1 knockdown cells into a xenograft model resulted in sensitivity to anti-PD1 treatment and an increase in the overall survival [99]. 

The regulating role of the gut microbiome in patient responses to immunotherapy has also been investigated in melanoma, NSCLC, renal cell carcinoma, and urothelial carcinoma [100]. In a fecal metagenomics study, Zheng et al. reported that four Lactobacillus species were most abundant in the anti-PD-1 therapy response group; these data illustrated the characteristic profile of the gut microbiome as a novel predictive index of early outcomes in HCC patients receiving anti-PD-1 [101]. A clinical trial analyzed the baseline levels of plasma cytokines or chemokines in 24 patients with HCC, and their responses to pembrolizumab noted that non-responders had a much lower TGF-β level [102]. A different prospective study of magnetic resonance elastography evaluation of patients with advanced HCC treated with pembrolizumab noted an increase in HCC stiffness at six weeks, which was correlated with OS [103].

## 6. The Future of Immunotherapy

Mouse models have played important roles in the identification of the complex molecular and cellular processes involved in HCC due, in part, to the oncogenic variation of human HCC and its heterogenous nature [99]. Ongoing human trials are underway to review different cancer immune therapies. Many therapies discussed above are still being studied currently. For example, the expression of phosphatase of regenerating liver-3 (PRL-3) was noted to be upregulated in a variety of tumor cells across 11 cancers. A humanized antibody targeting this TAA, PRL3-zumab, was demonstrated to promote recruitment of B cells, NK cells and macrophages, which suggests that this therapy might promote tumor killing by antibody-dependent, cell-mediated cytotoxicity [100]. 

## 7. Conclusions

Liver resection and transplantation are two surgical options that offer the best chance at long-term survival in patients with HCC. The majority of patients are, however, diagnosed at late stages and often are not candidates for curative surgical management. Moreover, traditional systemic treatment options for patients with advanced HCC have been limited, with modest response rates and poor long-term survival benefit. The heterogeneous etiology, complex tumor microenvironment, and immunomodulation of HCC require a specific treatment strategy with novel targeted therapies. Recent advances in molecular profiling have shed light on the understanding of the immunologic microenvironment of HCC. Since FDA approval of sorafenib, there has been a growing interest in the development of a new generation of systemic therapies. More recently, since the FDA approval of nivolumab as a second-line treatment for patients with advanced HCC, there has been an explosion of research exploring novel anti-immune checkpoint targets. The results of studies evaluating the efficacy of combination regimens of anti-angiogenesis agents with immunotherapy have been promising, with particular potential for effective therapy in patients with advanced HCC. Of note, limitations related to immunotherapy largely relate to the safety profile, as well as biomarker expression not always correlating with responsiveness to immune checkpoint therapy. As a result, biomarker expression measurement cannot be used as a reliable predictor of immunotherapy effectiveness. In addition, adoptive cell transfer therapy still requires long-term clinical data from larger cohorts to validate its safety and reliability. The complexity of the mechanisms responsible for HCC immune system dysregulation will require the development of more robust biomarkers to predict therapeutic response and optimize personalized treatment regimens.

## Figures and Tables

**Table 1:** Summary of clinical trials involving ICIs for the treatment of HCC.

NCT Number	Treatment [70]	Setting	Design
NCT01658878	Nivolumab	Second line in sorafenib pretreated patients	Phase I-II dose escalation and expansion
NCT01658878	Nivolumab + Ipilimumab	Second line in sorafenib pretreated patients	Phase I-II
NCT02576509	Nivolumab vs Sorafenib	First line treatment	Phase III
NCT02702414	Pembrolizumab	Second line in sorafenib pretreated patients	Phase II
NCT02702401	Pembrolizumab vs placebo	Second line treatment	Phase III
NCT03006926	Pembrolizumab + Lenvatinib	First line treatment	Phase Ib
NCT03289533	Avelumab + Axitinib	First line treatment	Phase Ib
NCT03510871	Nivolumab or Nivolumab + Ipilimumab	Perioperative treatment, resectable HCC	Phase II
NCT02715531	Atezolizumab + Bevacizumab vs Atezolizumab alone	First line treatment	Phase Ib
NCT03434379	Atezolizumab + Bevacizumab vs Sorafenib	First line treatment	Phase III
NCT01693562	Durvalumab	Mainly second line in sorafenib pretreated patients	Phase I-II
NCT02519348	Durvalumab + Tremelimumab	Mainly second line in sorafenib pretreated patients	Phase I
NCT02572687	Durvalumab + Ramucirumab	Second line treatment	Phase I
NCT01008358	Tremelimumab	Pretreated advanced HCC from HCV	Phase II
NCT01853618	Tremelimumab + ablation	Locally advanced HCC	Phase I-II
NCT02989922	Camrelizumab	Second line treatment	Phase II
NCT03463876	Camrelizumab + Apatinib	Advanced HCC	Phase II
NCT03092895	Camrelizumab + FOLFOX4 or GEMOX regimen	First line treatment	Phase II
NCT02407990	Tislelizumab	Sorafenib-refractory HCC	Phase I dose escalation and expansion

Abbreviations: ORR: overall response rate; mPFS: median progression-free survival; mOS: median overall survival; PR: partial response; SD: stable disease; HCV: hepatitis C virus; pCR: pathologic complete response; TACE: trans-arterial chemoembolization.

## Abbreviations

ACT: adoptive cell therapy; AJCC: American Joint Committee on Cancer; APC: antigen-presenting cells; ARG1: Arginase 1; BCLC: Barcelona Clinic Liver Cancer; CAR: chimeric antigen receptor; CIK: cytokine-induced killer; CTLA-4: cytotoxic T lymphocyte–associated antigen 4; CRS: cytokine release syndrome; DC: dendritic cells; DOR: duration of response; GPC-3: glypican-3; HAF: hypoxia-associated factor; HCC: hepatocellular carcinoma; hTERT: human telomerase reverse transcriptase; KIR: killer cell immunoglobulin-like receptors; iNOS: inducible nitric oxide synthase; mRECIST: modified Response Evaluation Criteria in Solid Tumors; lncRNA: long noncoding RNA; LC: Langerhans cells; MAGE-1: melanoma antigen gene-1; MDSC: myeloid-derived suppressor cells; NK: natural killer; ORR: objective response rates; OS: overall survival; PD-1: programmed cell death-1; PDL-1: programmed cell death ligand-1; PDGF: platelet-derived growth factor; pNLR: peritumoral neutrophil-to-lymphocyte ratio; PFS: progression-free survival; PRL-3: phosphatase of regenerating liver-3; RAF: rapidly accelerated fibrosarcoma; ROS: reactive oxygen species; TAA: tumor-associated antigens; TAM: tumor-associated macrophages; TAN: tumor-associated neutrophils; TACE: trans-arterial chemoembolization; TARE: trans-arterial radioembolization; TTP: time to progression; TCR: T cell receptor; TIL: tumor infiltrating; Tregs: regulatory T cells; TCR: T cell receptor; TLS: tumor lysis syndrome; TMB: tumor mutational burden; VEGF: vascular endothelial growth factor.

## References

[1] Townsend C.M., Beauchamp R.D., Mattox K.L., Evers B.M. (2017). Sabiston Textbook of Surgery.

[2] Brunicardi F.C., Andersen D.K., Billiar T.R., Dunn D.L., Hunter J.G., Kao L., Matthews J.B., Pollock R.E. (2019). Chapter 1: Leadership in Surgery. Schwartz Principles of Surgery.

[3] Yang J.D., Hainaut P., Gores G.J., Amadou A., Plymoth A., Roberts L.R. (2019). A global view of hepatocellular carcinoma: Trends, risk, prevention and management. Nat. Rev. Gastroenterol. Hepatol..

[4] Pinato D.J., Sharma R., Allara E., Yen C., Arizumi T., Kubota K., Bettinger D., Jang J.W., Smirne C., Kim Y.W. (2017). The ALBI grade provides objective hepatic reserve estimation across each BCLC stage of hepatocellular carcinoma. J. Hepatol..

[5] Couri T., Pillai A. (2019). Goals and targets for personalized therapy for HCC. Hepatol. Int..

[6] Heinrich B., Czauderna C., Marquardt J.U. (2018). Immunotherapy of Hepatocellular Carcinoma. Oncol. Res. Treat..

[7] Abd El Aziz M.A., Facciorusso A., Nayfeh T., Saadi S., Elnaggar M., Cotsoglou C., Sacco R. (2020). Immune Checkpoint Inhibitors for Unresectable Hepatocellular Carcinoma. Vaccines.

[8] Llovet J.M., Ricci S., Mazzaferro V., Hilgard P., Gane E., Blanc J.F., de Oliveira A.C., Santoro A., Raoul J.L., Forner A. (2008). Sorafenib in advanced hepatocellular carcinoma. N. Engl. J. Med..

[9] Wilhelm S.M., Adnane L., Newell P., Villanueva A., Llovet J.M., Lynch M. (2008). Preclinical overview of sorafenib, a multikinase inhibitor that targets both Raf and VEGF and PDGF receptor tyrosine kinase signaling. Mol. Cancer Ther..

[10] Tovoli F., De Lorenzo S., Trevisani F. (2020). Immunotherapy with Checkpoint Inhibitors for Hepatocellular Carcinoma: Where Are We Now?. Vaccines.

[11] Tahmasebi Birgani M., Carloni V. (2017). Tumor Microenvironment, a Paradigm in Hepatocellular Carcinoma Progression and Therapy. Int. J. Mol. Sci..

[12] Wang G., Wang Q., Liang N., Xue H., Yang T., Chen X., Qiu Z., Zeng C., Sun T., Yuan W. (2020). Oncogenic driver genes and tumor microenvironment determine the type of liver cancer. Cell Death Dis..

[13] Fu Y., Liu S., Zeng S., Shen H. (2019). From bench to bed: The tumor immune microenvironment and current immunotherapeutic strategies for hepatocellular carcinoma. J. Exp. Clin. Cancer Res..

[14] Patel K., Lamm R., Altshuler P., Dang H., Shah A.P. (2020). Hepatocellular Carcinoma-The Influence of Immunoanatomy and the Role of Immunotherapy. Int. J. Mol. Sci..

[15] Keenan B.P., Fong L., Kelley R.K. (2019). Immunotherapy in hepatocellular carcinoma: The complex interface between inflammation, fibrosis, and the immune response. J. Immunother. Cancer.

[16] Wan S., Zhao E., Kryczek I., Vatan L., Sadovskaya A., Ludema G., Simeone D.M., Zou W., Welling T.H. (2014). Tumor-associated macrophages produce interleukin 6 and signal via STAT3 to promote expansion of human hepatocellular carcinoma stem cells. Gastroenterology.

[17] Yin Z., Ma T., Lin Y., Lu X., Zhang C., Chen S., Jian Z. (2018). IL-6/STAT3 pathway intermediates M1/M2 macrophage polarization during the development of hepatocellular carcinoma. J. Cell. Biochem..

[18] Yao R.R., Li J.H., Zhang R., Chen R.X., Wang Y.H. (2018). M2-polarized tumor-associated macrophages facilitated migration and epithelial-mesenchymal transition of HCC cells via the TLR4/STAT3 signaling pathway. World J. Surg. Oncol..

[19] Masucci M.T., Minopoli M., Carriero M.V. (2019). Tumor Associated Neutrophils. Their Role in Tumorigenesis, Metastasis, Prognosis and Therapy. Front. Oncol..

[20] Byun J.S., Yi H.S. (2017). Hepatic Immune Microenvironment in Alcoholic and Nonalcoholic Liver Disease. BioMed Res. Int..

[21] He G., Zhang H., Zhou J., Wang B., Chen Y., Kong Y., Xie X., Wang X., Fei R., Wei L. (2015). Peritumoural neutrophils negatively regulate adaptive immunity via the PD-L1/PD-1 signalling pathway in hepatocellular carcinoma. J. Exp. Clin. Cancer Res. CR.

[22] Dardalhon V., Anderson A.C., Karman J., Apetoh L., Chandwaskar R., Lee D.H., Cornejo M., Nishi N., Yamauchi A., Quintana F.J. (2010). Tim-3/galectin-9 pathway: Regulation of Th1 immunity through promotion of CD11b+Ly-6G+ myeloid cells. J. Immunol. (Baltimore Md. 1950).

[23] Hoechst B., Ormandy L.A., Ballmaier M., Lehner F., Krüger C., Manns M.P., Greten T.F., Korangy F. (2008). A new population of myeloid-derived suppressor cells in hepatocellular carcinoma patients induces CD4(+)CD25(+)Foxp3(+) T cells. Gastroenterology.

[24] Jiang R., Tang J., Chen Y., Deng L., Ji J., Xie Y., Wang K., Jia W., Chu W.M., Sun B. (2017). The long noncoding RNA lnc-EGFR stimulates T-regulatory cells differentiation thus promoting hepatocellular carcinoma immune evasion. Nat. Commun..

[25] Park R., Eshrat F., Al-Jumayli M., Saeed A., Saeed A. (2020). Immuno-Oncotherapeutic Approaches in Advanced Hepatocellular Carcinoma. Vaccines.

[26] Zhu W., Peng Y., Wang L., Hong Y., Jiang X., Li Q., Liu H., Huang L., Wu J., Celis E. (2018). Identification of α-fetoprotein-specific T-cell receptors for hepatocellular carcinoma immunotherapy. Hepatology (Baltim Md.).

[27] Tada F., Abe M., Hirooka M., Ikeda Y., Hiasa Y., Lee Y., Jung N.C., Lee W.B., Lee H.S., Bae Y.S. (2012). Phase I/II study of immunotherapy using tumor antigen-pulsed dendritic cells in patients with hepatocellular carcinoma. Int. J. Oncol..

[28] Lu Z., Zuo B., Jing R., Gao X., Rao Q., Liu Z., Qi H., Guo H., Yin H. (2017). Dendritic cell-derived exosomes elicit tumor regression in autochthonous hepatocellular carcinoma mouse models. J. Hepatol..

[29] Tsuchiya N., Yoshikawa T., Fujinami N., Saito K., Mizuno S., Sawada Y., Endo I., Nakatsura T. (2017). Immunological efficacy of glypican-3 peptide vaccine in patients with advanced hepatocellular carcinoma. Oncoimmunology.

[30] Xu F., Jin T., Zhu Y., Dai C. (2018). Immune checkpoint therapy in liver cancer. J. Exp. Clin. Cancer Res. CR.

[31] Sharpe A.H., Pauken K.E. (2018). The diverse functions of the PD1 inhibitory pathway. Nat. Rev. Immunol..

[32] Wang X., He Q., Shen H., Xia A., Tian W., Yu W., Sun B. (2019). TOX promotes the exhaustion of antitumor CD8(+) T cells by preventing PD1 degradation in hepatocellular carcinoma. J. Hepatol..

[33] Zhao Y., Yang W., Huang Y., Cui R., Li X., Li B. (2018). Evolving Roles for Targeting CTLA-4 in Cancer Immunotherapy. Cell. Physiol. Biochem. Int. J. Exp. Cell. Physiol. Biochem. Pharmacol..

[34] Agdashian D., ElGindi M., Xie C., Sandhu M., Pratt D., Kleiner D.E., Figg W.D., Rytlewski J.A., Sanders C., Yusko E.C. (2019). The effect of anti-CTLA4 treatment on peripheral and intra-tumoral T cells in patients with hepatocellular carcinoma. Cancer Immunol. Immunother. CII.

[35] Principe N., Kidman J., Goh S., Tilsed C.M., Fisher S.A., Fear V.S., Forbes C.A., Zemek R.M., Chopra A., Watson M. (2020). Tumor Infiltrating Effector Memory Antigen-Specific CD8(+) T Cells Predict Response to Immune Checkpoint Therapy. Front. Immunol..

[36] Xie Y., Xiang Y., Sheng J., Zhang D., Yao X., Yang Y., Zhang X. (2018). Immunotherapy for Hepatocellular Carcinoma: Current Advances and Future Expectations. J. Immunol. Res..

[37] June C.H., O’Connor R.S., Kawalekar O.U., Ghassemi S., Milone M.C. (2018). CAR T cell immunotherapy for human cancer. Science (N. Y.).

[38] Guo X., Jiang H., Shi B., Zhou M., Zhang H., Shi Z., Du G., Luo H., Wu X., Wang Y. (2018). Disruption of PD-1 Enhanced the Anti-tumor Activity of Chimeric Antigen Receptor T Cells Against Hepatocellular Carcinoma. Front. Pharmacol..

[39] Li D., Li N., Zhang Y.F., Fu H., Feng M., Schneider D., Su L., Wu X., Zhou J., Mackay S. (2020). Persistent Polyfunctional Chimeric Antigen Receptor T Cells That Target Glypican 3 Eliminate Orthotopic Hepatocellular Carcinomas in Mice. Gastroenterology.

[40] Sun B., Yang D., Dai H., Liu X., Jia R., Cui X., Li W., Cai C., Xu J., Zhao X. (2019). Eradication of Hepatocellular Carcinoma by NKG2D-Based CAR-T Cells. Cancer Immunol. Res..

[41] Li K., Qian S., Huang M., Chen M., Peng L., Liu J., Xu W., Xu J. (2021). Development of GPC3 and EGFR-dual-targeting chimeric antigen receptor-T cells for adoptive T cell therapy. Am. J. Transl. Res..

[42] Chen C., Li K., Jiang H., Song F., Gao H., Pan X., Shi B., Bi Y., Wang H., Wang H. (2017). Development of T cells carrying two complementary chimeric antigen receptors against glypican-3 and asialoglycoprotein receptor 1 for the treatment of hepatocellular carcinoma. Cancer Immunol. Immunother. CII.

[43] Liu H., Xu Y., Xiang J., Long L., Green S., Yang Z., Zimdahl B., Lu J., Cheng N., Horan L.H. (2017). Targeting Alpha-Fetoprotein (AFP)-MHC Complex with CAR T-Cell Therapy for Liver Cancer. Clin. Cancer Res. Off. J. Am. Assoc. Cancer Res..

[44] Zou F., Tan J., Liu T., Liu B., Tang Y., Zhang H., Li J. (2021). The CD39(+) HBV surface protein-targeted CAR-T and personalized tumor-reactive CD8(+) T cells exhibit potent anti-HCC activity. Mol. Ther. J. Am. Soc. Gene Ther..

[45] Zhang R.Y., Wei D., Liu Z.K., Yong Y.L., Wei W., Zhang Z.Y., Lv J.J., Zhang Z., Chen Z.N., Bian H. (2019). Doxycycline Inducible Chimeric Antigen Receptor T Cells Targeting CD147 for Hepatocellular Carcinoma Therapy. Front. Cell Dev. Biol..

[46] Tseng H.C., Xiong W., Badeti S., Yang Y., Ma M., Liu T., Ramos C.A., Dotti G., Fritzky L., Jiang J.G. (2020). Efficacy of anti-CD147 chimeric antigen receptors targeting hepatocellular carcinoma. Nat. Commun..

[47] Shi D., Shi Y., Kaseb A.O., Qi X., Zhang Y., Chi J., Lu Q., Gao H., Jiang H., Wang H. (2020). Chimeric Antigen Receptor-Glypican-3 T-Cell Therapy for Advanced Hepatocellular Carcinoma: Results of Phase I Trials. Clin. Cancer Res. Off. J. Am. Assoc. Cancer Res..

[48] Meng Y., Yu Z., Wu Y., Du T., Chen S., Meng F., Su N., Ma Y., Li X., Sun S. (2017). Cell-based immunotherapy with cytokine-induced killer (CIK) cells: From preparation and testing to clinical application. Hum. Vaccines Immunother..

[49] Wang F.S., Liu M.X., Zhang B., Shi M., Lei Z.Y., Sun W.B., Du Q.Y., Chen J.M. (2002). Antitumor activities of human autologous cytokine-induced killer (CIK) cells against hepatocellular carcinoma cells in vitro and in vivo. World J. Gastroenterol..

[50] Zhang Y.S., Yuan F.J., Jia G.F., Zhang J.F., Hu L.Y., Huang L., Wang J., Dai Z.Q. (2005). CIK cells from patients with HCC possess strong cytotoxicity to multidrug-resistant cell line Bel-7402/R. World J. Gastroenterol..

[51] Rong X.X., Wei F., Lin X.L., Qin Y.J., Chen L., Wang H.Y., Shen H.F., Jia L.T., Xie R.Y., Lin T.Y. (2016). Recognition and killing of cancer stem-like cell population in hepatocellular carcinoma cells by cytokine-induced killer cells via NKG2d-ligands recognition. Oncoimmunology.

[52] Yang T., Zhang W., Wang L., Xiao C., Wang L., Gong Y., Huang D., Guo B., Li Q., Xiang Y. (2018). Co-culture of dendritic cells and cytokine-induced killer cells effectively suppresses liver cancer stem cell growth by inhibiting pathways in the immune system. BMC Cancer.

[53] González-Carmona M.A., Märten A., Hoffmann P., Schneider C., Sievers E., Schmidt-Wolf I.G., Sauerbruch T., Caselmann W.H. (2006). Patient-derived dendritic cells transduced with an a-fetoprotein-encoding adenovirus and co-cultured with autologous cytokine-induced lymphocytes induce a specific and strong immune response against hepatocellular carcinoma cells. Liver Int. Off. J. Int. Assoc. Study Liver.

[54] Xu K., Meng Z., Mu X., Sun B., Chai Y. (2020). One Single Site Clinical Study: To Evaluate the Safety and Efficacy of Immunotherapy With Autologous Dendritic Cells, Cytokine-Induced Killer Cells in Primary Hepatocellular Carcinoma Patients. Front. Oncol..

[55] Cao J., Kong F.H., Liu X., Wang X.B. (2019). Immunotherapy with dendritic cells and cytokine-induced killer cells for hepatocellular carcinoma: A meta-analysis. World J. Gastroenterol..

[56] Chang B., Shen L., Wang K., Jin J., Huang T., Chen Q., Li W., Wu P. (2018). High number of PD-1 positive intratumoural lymphocytes predicts survival benefit of cytokine-induced killer cells for hepatocellular carcinoma patients. Liver Int. Off. J. Int. Assoc. Study Liver.

[57] Pan Q.Z., Liu Q., Zhou Y.Q., Zhao J.J., Wang Q.J., Li Y.Q., Tang Y., Gu J.M., He J., Chen S.P. (2020). CIK cell cytotoxicity is a predictive biomarker for CIK cell immunotherapy in postoperative patients with hepatocellular carcinoma. Cancer Immunol. Immunother. CII.

[58] Pan C.C., Huang Z.L., Li W., Zhao M., Zhou Q.M., Xia J.C., Wu P.H. (2010). Serum alpha-fetoprotein measurement in predicting clinical outcome related to autologous cytokine-induced killer cells in patients with hepatocellular carcinoma undergone minimally invasive therapy. Chin. J. Cancer.

[59] Liu Q., Tian Y., Li Y., Zhang W., Cai W., Liu Y., Ren Y., Liang Z., Zhou P., Zhang Y. (2020). In vivo therapeutic effects of affinity-improved-TCR engineered T-cells on HBV-related hepatocellular carcinoma. J. Immunother. Cancer.

[60] Docta R.Y., Ferronha T., Sanderson J.P., Weissensteiner T., Pope G.R., Bennett A.D., Pumphrey N.J., Ferjentsik Z., Quinn L.L., Wiedermann G.E. (2019). Tuning T-Cell Receptor Affinity to Optimize Clinical Risk-Benefit When Targeting Alpha-Fetoprotein-Positive Liver Cancer. Hepatology (Baltim. Md.).

[61] Goyal L., Frigault M., Meyer T., Feun L.G., Bruix J., El-Khoueiry A., Hausner P., Sangro B., Pierce T.T., Norry E. (2019). Initial safety of AFP SPEAR T-cells in patients with advanced hepatocellular carcinoma. AACR.

[62] Luo X., Cui H., Cai L., Zhu W., Yang W.C., Patrick M., Zhu S., Huang J., Yao X., Yao Y. (2020). Selection of a Clinical Lead TCR Targeting Alpha-Fetoprotein-Positive Liver Cancer Based on a Balance of Risk and Benefit. Front. Immunol..

[63] Chew V., Tow C., Teo M., Wong H.L., Chan J., Gehring A., Loh M., Bolze A., Quek R., Lee V.K. (2010). Inflammatory tumour microenvironment is associated with superior survival in hepatocellular carcinoma patients. J. Hepatol..

[64] Zhou G., Sprengers D., Boor P.P.C., Doukas M., Schutz H., Mancham S., Pedroza-Gonzalez A., Polak W.G., de Jonge J., Gaspersz M. (2017). Antibodies Against Immune Checkpoint Molecules Restore Functions of Tumor-Infiltrating T Cells in Hepatocellular Carcinomas. Gastroenterology.

[65] Jiang S.S., Tang Y., Zhang Y.J., Weng D.S., Zhou Z.G., Pan K., Pan Q.Z., Wang Q.J., Liu Q., He J. (2015). A phase I clinical trial utilizing autologous tumor-infiltrating lymphocytes in patients with primary hepatocellular carcinoma. Oncotarget.

[66] Gross G., Eshhar Z. (2016). Therapeutic Potential of T Cell Chimeric Antigen Receptors (CARs) in Cancer Treatment: Counteracting Off-Tumor Toxicities for Safe CAR T Cell Therapy. Annu. Rev. Pharm. Toxicol..

[67] Teachey D.T., Lacey S.F., Shaw P.A., Melenhorst J.J., Maude S.L., Frey N., Pequignot E., Gonzalez V.E., Chen F., Finklestein J. (2016). Identification of Predictive Biomarkers for Cytokine Release Syndrome after Chimeric Antigen Receptor T-cell Therapy for Acute Lymphoblastic Leukemia. Cancer Discov..

[68] Xu X.J., Tang Y.M. (2014). Cytokine release syndrome in cancer immunotherapy with chimeric antigen receptor engineered T cells. Cancer Lett..

[69] Zhu A.X., Kudo M., Assenat E., Cattan S., Kang Y.K., Lim H.Y., Poon R.T., Blanc J.F., Vogel A., Chen C.L. (2014). Effect of everolimus on survival in advanced hepatocellular carcinoma after failure of sorafenib: The EVOLVE-1 randomized clinical trial. JAMA.

[70] Ho W.J., Sharma G., Zhu Q., Stein-O’Brien G., Durham J., Anders R., Popovic A., Mo G., Kamel I., Weiss M. (2020). Integrated immunological analysis of a successful conversion of locally advanced hepatocellular carcinoma to resectability with neoadjuvant therapy. J. Immunother. Cancer.

[71] Yau T., Kang Y.K., Kim T.Y., El-Khoueiry A.B., Santoro A., Sangro B., Melero I., Kudo M., Hou M.M., Matilla A. (2020). Efficacy and Safety of Nivolumab Plus Ipilimumab in Patients With Advanced Hepatocellular Carcinoma Previously Treated With Sorafenib: The CheckMate 040 Randomized Clinical Trial. JAMA Oncol..

[72] He A.R., Yau T., Hsu C., Kang Y.-K., Kim T.-Y., Santoro A., Sangro B., Melero I., Kudo M., Hou M.-M. (2020). Nivolumab (NIVO) + ipilimumab (IPI) combination therapy in patients (pts) with advanced hepatocellular carcinoma (aHCC): Subgroup analyses from CheckMate 040. J. Clin. Oncol..

[73] Kelley R.K., Abou-Alfa G.K., Bendell J.C., Kim T.-Y., Borad M.J., Yong W.-P., Morse M., Kang Y.-K., Rebelatto M., Makowsky M. (2017). Phase I/II study of durvalumab and tremelimumab in patients with unresectable hepatocellular carcinoma (HCC): Phase I safety and efficacy analyses. Am. Soc. Clin. Oncol..

[74] Pinato D.J., Guerra N., Fessas P., Murphy R., Mineo T., Mauri F.A., Mukherjee S.K., Thursz M., Wong C.N., Sharma R. (2020). Immune-based therapies for hepatocellular carcinoma. Oncogene.

[75] Vey N., Karlin L., Sadot-Lebouvier S., Broussais F., Berton-Rigaud D., Rey J., Charbonnier A., Marie D., André P., Paturel C. (2018). A phase 1 study of lirilumab (antibody against killer immunoglobulin-like receptor antibody KIR2D; IPH2102) in patients with solid tumors and hematologic malignancies. Oncotarget.

[76] André P., Denis C., Soulas C., Bourbon-Caillet C., Lopez J., Arnoux T., Bléry M., Bonnafous C., Gauthier L., Morel A. (2018). Anti-NKG2A mAb Is a Checkpoint Inhibitor that Promotes Anti-tumor Immunity by Unleashing Both T and NK Cells. Cell.

[77] Jin H., Wang C., Jin G., Ruan H., Gu D., Wei L., Wang H., Wang N., Arunachalam E., Zhang Y. (2017). Regulator of Calcineurin 1 Gene Isoform 4, Down-regulated in Hepatocellular Carcinoma, Prevents Proliferation, Migration, and Invasive Activity of Cancer Cells and Metastasis of Orthotopic Tumors by Inhibiting Nuclear Translocation of NFAT1. Gastroenterology.

[78] Tang W., Chen Z., Zhang W., Cheng Y., Zhang B., Wu F., Wang Q., Wang S., Rong D., Reiter F.P. (2020). The mechanisms of sorafenib resistance in hepatocellular carcinoma: Theoretical basis and therapeutic aspects. Signal Transduct. Target. Ther..

[79] Yau T., Park J., Finn R., Cheng A.-L., Mathurin P., Edeline J., Kudo M., Han K.-H., Harding J., Merle P.J.A. (2019). CheckMate 459: A randomized, multi-center phase III study of nivolumab (NIVO) vs sorafenib (SOR) as first-line (1L) treatment in patients (pts) with advanced hepatocellular carcinoma (aHCC). Ann. Oncol..

[80] Finn R.S., Qin S., Ikeda M., Galle P.R., Ducreux M., Kim T.Y., Kudo M., Breder V., Merle P., Kaseb A.O. (2020). Atezolizumab plus Bevacizumab in Unresectable Hepatocellular Carcinoma. N. Engl. J. Med..

[81] Llovet J.M., Kudo M., Cheng A.-L., Finn R.S., Galle P.R., Kaneko S., Meyer T., Qin S., Dutcus C.E., Chen E. (2019). Lenvatinib (len) plus pembrolizumab (pembro) for the first-line treatment of patients (pts) with advanced hepatocellular carcinoma (HCC): Phase 3 LEAP-002 study. Am. Soc. Clin. Oncol..

[82] Kudo M., Motomura K., Wada Y., Inaba Y., Sakamoto Y., Kurosaki M., Umeyama Y., Kamei Y., Yoshimitsu J., Fujii Y. (2019). First-line avelumab+ axitinib in patients with advanced hepatocellular carcinoma: Results from a phase 1b trial (VEGF Liver 100). Am. Soc. Clin. Oncol..

[83] Greten T.F., Mauda-Havakuk M., Heinrich B., Korangy F., Wood B.J. (2019). Combined locoregional-immunotherapy for liver cancer. J. Hepatol..

[84] Duffy A.G., Ulahannan S.V., Makorova-Rusher O., Rahma O., Wedemeyer H., Pratt D., Davis J.L., Hughes M.S., Heller T., ElGindi M. (2017). Tremelimumab in combination with ablation in patients with advanced hepatocellular carcinoma. J. Hepatol..

[85] Pinato D.J., Cole T., Bengsch B., Tait P., Sayed A.A., Abomeli F., Gramenitskaya D., Allara E., Thomas R., Ward C.J.A. (2019). A phase Ib study of pembrolizumab following trans-arterial chemoembolization (TACE) in hepatocellular carcinoma (HCC): PETAL. Ann. Oncol..

[86] Hoseini S.S., Cheung N.V. (2017). Immunotherapy of hepatocellular carcinoma using chimeric antigen receptors and bispecific antibodies. Cancer Lett..

[87] Ishiguro T., Sano Y., Komatsu S.I., Kamata-Sakurai M., Kaneko A., Kinoshita Y., Shiraiwa H., Azuma Y., Tsunenari T., Kayukawa Y. (2017). An anti-glypican 3/CD3 bispecific T cell-redirecting antibody for treatment of solid tumors. Sci. Transl. Med..

[88] El-Khoueiry A.B., Sangro B., Yau T., Crocenzi T.S., Kudo M., Hsu C., Kim T.Y., Choo S.P., Trojan J., Welling T.H.R. (2017). Nivolumab in patients with advanced hepatocellular carcinoma (CheckMate 040): An open-label, non-comparative, phase 1/2 dose escalation and expansion trial. Lancet (Lond. Engl.).

[89] Zhu A.X., Finn R.S., Edeline J., Cattan S., Ogasawara S., Palmer D., Verslype C., Zagonel V., Fartoux L., Vogel A. (2018). Pembrolizumab in patients with advanced hepatocellular carcinoma previously treated with sorafenib (KEYNOTE-224): A non-randomised, open-label phase 2 trial. Lancet. Oncol..

[90] Chang B., Huang T., Wei H., Shen L., Zhu D., He W., Chen Q., Zhang H., Li Y., Huang R. (2019). The correlation and prognostic value of serum levels of soluble programmed death protein 1 (sPD-1) and soluble programmed death-ligand 1 (sPD-L1) in patients with hepatocellular carcinoma. Cancer Immunol. Immunother. CII.

[91] Ho W.J., Danilova L., Lim S.J., Verma R., Xavier S., Leatherman J.M., Sztein M.B., Fertig E.J., Wang H., Jaffee E. (2020). Viral status, immune microenvironment and immunological response to checkpoint inhibitors in hepatocellular carcinoma. J. Immunother. Cancer.

[92] Goodman A.M., Kato S., Bazhenova L., Patel S.P., Frampton G.M., Miller V., Stephens P.J., Daniels G.A., Kurzrock R. (2017). Tumor Mutational Burden as an Independent Predictor of Response to Immunotherapy in Diverse Cancers. Mol. Cancer Ther..

[93] Yarchoan M., Hopkins A., Jaffee E.M. (2017). Tumor Mutational Burden and Response Rate to PD-1 Inhibition. N. Engl. J. Med..

[94] Ang C., Klempner S.J., Ali S.M., Madison R., Ross J.S., Severson E.A., Fabrizio D., Goodman A., Kurzrock R., Suh J. (2019). Prevalence of established and emerging biomarkers of immune checkpoint inhibitor response in advanced hepatocellular carcinoma. Oncotarget.

[95] Kawaoka T., Ando Y., Yamauchi M., Suehiro Y., Yamaoka K., Kosaka Y., Fuji Y., Uchikawa S., Morio K., Fujino H. (2020). Incidence of microsatellite instability-high hepatocellular carcinoma among Japanese patients and response to pembrolizumab. Hepatol. Res. Off. J. Jpn. Soc. Hepatol..

[96] Dudley J.C., Lin M.T., Le D.T., Eshleman J.R. (2016). Microsatellite Instability as a Biomarker for PD-1 Blockade. Clin. Cancer Res. Off. J. Am. Assoc. Cancer Res..

[97] Le D.T., Durham J.N., Smith K.N., Wang H., Bartlett B.R., Aulakh L.K., Lu S., Kemberling H., Wilt C., Luber B.S. (2017). Mismatch repair deficiency predicts response of solid tumors to PD-1 blockade. Science (N. Y.).

[98] Harding J.J., Nandakumar S., Armenia J., Khalil D.N., Albano M., Ly M., Shia J., Hechtman J.F., Kundra R., El Dika I. (2019). Prospective Genotyping of Hepatocellular Carcinoma: Clinical Implications of Next-Generation Sequencing for Matching Patients to Targeted and Immune Therapies. Clin. Cancer Res. Off. J. Am. Assoc. Cancer Res..

[99] Zhang P.F., Gao C., Huang X.Y., Lu J.C., Guo X.J., Shi G.M., Cai J.B., Ke A.W. (2020). Cancer cell-derived exosomal circUHRF1 induces natural killer cell exhaustion and may cause resistance to anti-PD1 therapy in hepatocellular carcinoma. Mol. Cancer.

[100] Sepich-Poore G.D., Zitvogel L., Straussman R., Hasty J., Wargo J.A., Knight R. (2021). The microbiome and human cancer. Science (N. Y.).

[101] Zheng Y., Wang T., Tu X., Huang Y., Zhang H., Tan D., Jiang W., Cai S., Zhao P., Song R. (2019). Gut microbiome affects the response to anti-PD-1 immunotherapy in patients with hepatocellular carcinoma. J. Immunother. Cancer.

[102] Feun L.G., Li Y.Y., Wu C., Wangpaichitr M., Jones P.D., Richman S.P., Madrazo B., Kwon D., Garcia-Buitrago M., Martin P. (2019). Phase 2 study of pembrolizumab and circulating biomarkers to predict anticancer response in advanced, unresectable hepatocellular carcinoma. Cancer.

[103] Qayyum A., Hwang K.P., Stafford J., Verma A., Maru D.M., Sandesh S., Sun J., Pestana R.C., Avritscher R., Hassan M.M. (2019). Immunotherapy response evaluation with magnetic resonance elastography (MRE) in advanced HCC. J. Immunother. Cancer.

